# Platelet-Rich Plasma for Elbow Pathologies: a Descriptive Review of Current Literature

**DOI:** 10.1007/s12178-018-9520-1

**Published:** 2018-09-25

**Authors:** Adam Kwapisz, Sharad Prabhakar, Riccardo Compagnoni, Aleksandra Sibilska, Pietro Randelli

**Affiliations:** 1 Clinic of Orthopedics and Pediatric Orthopedics, Medical University of Lodz, Lodz, Poland; 2 Department of Orthopedics Post Graduate Institute of Medical Education and Research, Chandigarh, India; 3 Laboratory of Applied Biomechanics, Department of Biomedical Sciences for Health, UniversitàdegliStudi di Milano, Via Mangiagalli 31, 20133 Milan, Italy; 4 1 Clinica Ortopedica, ASST Centro Specialistico Ortopedico Traumatologico Gaetano Pini-CTO, Piazza Cardinal Ferrari 1, 20122 Milan, Italy

**Keywords:** Platelets-rich plasma, Elbow, Tendinopathy, Tennis elbow, Golfer’s elbow

## Abstract

**Purpose of Review:**

Platelet-rich plasma is used in many orthopedic pathologies such as tendinopathies and ligament injuries. The clinical results reported in the scientific literature are, however, confusing. The aim of this manuscript is to provide a narrative literature review regarding the role of PRP in the most common elbow soft tissue pathologies.

**Recent Findings:**

The response to PRP seems to be favorable when compared to steroid injection for pain management and for patient-reported outcomes in lateral epicondylitis. PRP injection does not seem to have the potential complications associated with a steroid injection such as skin atrophy, discoloration, and secondary tendon tears. Only a few manuscripts comparing the results of PRP treatment with either extracorporeal shockwave (ESW), dry needling, or even surgical treatments in lateral epicondylitis exist. The use of PRP in other elbow pathologies such as golfer’s elbow, ulnar collateral ligament injury, and distal biceps and triceps pathology is examined in few studies, with unclear recommendations.

**Summary:**

Regarding elbow pathologies, PRP injections in tennis elbow seems to be the best-studied intervention. A major limitation in these studies is the significant heterogeneity in the methods used for preparing PRP, for example employing leukocyte-rich, leukocyte-poor preparations, PRP with or without activation, which makes the results of the studies difficult to compare. Results of this review show that more studies on larger cohorts, with comparable formulations, and with longer follow-up are required to give optimal suggestions concerning the use of PRP in elbow pathologies.

## Introduction

Platelet-rich plasma (PRP) is an autologous mixture of platelets and growth factors produced by centrifugation of whole blood [1]. PRP may enhance soft tissue regeneration processes by releasing platelet-derived growth factors, cytokines, and other proteins capable of stimulating and modulating the inflammatory response [2–4]. Chen et al. in their meta-analysis suggested that PRP is a safe and efficacious way of supporting tendon and ligament healing [1]. In vitro studies have shown that human tenocyte proliferation increases when cultured in PRP suggesting that PRP mediates the anabolic effect of growth factors enhancing tendon matrix regeneration. An increased TGF beta concentration has been correlated in many studies with the clinical efficacy of PRP [5–7].

In contrast to the basic science data, the clinical efficacy of PRP has been reported with conflicting result, including several systematic reviews and meta-analyses [8]. PRP has been used in many soft tissue pathologies such as tendinopathies and ligament injuries. Nevertheless, it remains unclear especially in chronic elbow conditions whether PRP should be recommended as a treatment option before performing a surgical treatment [1, 8–10, 11•]. The aim of this manuscript is to provide a narrative literature review regarding the role of PRP in the most common elbow soft tissue pathologies.

## Lateral Epicondylitis

Lateral epicondylitis, initially described in 1883 as “lawn-tennis elbow” and later named “tennis elbow” (TE), is the most commonly diagnosed elbow condition [12•, 13]. Epidemiological studies describe a prevalence of 1–3% in general population, with some reports describing data up to 10% in women [14, 15]. While TE has been extensively described by literature, its pathophysiology remains obscure. Historically, it was believed to be an onset of inflammation, but recent studies have revealed a paucity of inflammatory cells [16]. The most commonly reported mechanism is extensor carpi radialis brevis (ECRB) tendon micro-tearing and degeneration due to repetitive overload [16]. Nirschl et al. described the pathology as an angiofibroblastic tendinosis [17]. More recently, postero-lateral micro-instability has been suggested as a possible etiology for TE [18].

Different treatment approaches have been described for TE, starting from rest, bracing, eccentric muscle strengthening, and activity modification to second-line treatments like extracorporeal shockwave therapy, botulinum toxin injection, dry needling, autologous whole blood injection, and PRP. Surgical treatment is usually reserved after failure of conservative management. Steroid injections used to be considered a gold standard in TE management and are actually one of the most commonly performed treatment, but data from the literature do not support this approach as curative [16].

Arirachakaran et al. (Table 1) performed meta-analysis comparing PRP to autologous blood and steroid injection. The response to PRP was favorable when compared to steroid injection for pain management and for patient-reported outcomes. Furthermore, it was reported that PRP injection did not have the complications associated with a steroid injection such as skin atrophy, discoloration, and secondary tendon tears [16]. Two original papers, however, reported higher incidence of local pain after PRP administration [19•, 20•]. Gosens and Peerbooms in a randomized control trial described that steroid injection may give better pain relief in the first months, but after 2 years of follow-up the PRP group reported superior results. Patients who had steroid injections had clinical scores comparable to their baseline at 26 weeks follow-up [12•, 21•]. Similar conclusions were obtained in a meta-analysis by Mi et al. that found better outcomes with PRP at more than 6 months follow-up even though steroid injections gave better scores initially [22]. Comparable findings were also described by Gautam et al. when the Visual Analogue Scale (VAS), Disabilities of the Arm, Shoulder and Hand (DASH), and modified Mayo score were measured after 6 months. [23•] Yadav et al. and Behera et al. reported after 3 months follow-up significant improvement in pain and function in a PRP group. [24•, 25•] The randomized prospective study published by Palacio et al. reported that there was no significant difference in patients’ improvement when treated with PRP, dexamethasone, and neocaine [27•]. Two papers have also reported no significant difference in pain scores between PRP and steroids [22, 26•]. The lack of standardization of PRP preparations, described by Mishra et al., could partially justify the differences in reported outcomes among the studies [28]. In a multicenter randomized controlled trial, Mishra et al. compared the results of extensor tendon needling alone or in association with PRP injections. No significant differences were found at 12 weeks, but clinically meaningful improvements were found in patients treated with leukocyte-enriched PRP compared with an active control group at 24 weeks [29].

**Table 1:** Overview of the selected studies. (ABI – Autologus blood injection, PRP – Platelet Rich Plasma)

Author	Year	Study design	Comments
Mi et al	2017	Meta-analysis of randomized clinical trials	+ for PRP at 6 months (Steroid group had better initial results which declined subsequently)
Arirachakaran et al	2016	Network Meta-analysis and systematic review PRP vs steroid vs ABI	+ for PRP
Palacio et al	2016	PRP vs steroid	Equivocal
Gautam et al	2015	PRP vs steroid	+ for both PRP and steroid initially (Steroid group worsened after 6 months)
Khaliq A	2015	PRP vs steroid	+ for PRP
Lebiedziński et al	2015	PRP vs steroid	+ for PRP at one year
Yadav R	2015	PRP vs steroid	+ for PRP at 3 months
Behera P	2015	PRP vs steroid	+ for PRP at 3 months (Steroid group had better initial results which declined subsequently)
Krogh et al	2013	PRP vs steroids vs saline	Equivocal
Mishra et al	2012	PRP vs controls	+ for PRP at 6 months
Gosens et al	2011	PRP vs steroids	+ for PRP at 2 years (Steroid group had better initial results which declined subsequently)
Peerbooms	2010	PRP vs steroid injection	+ for PRP at one year (Steroid group had better initial results which declined subsequently)
Raeissadat et al	2014	PRP vs ABI	Equivocal
Thanasas	2011	PRP vs ABI	Equivocal (PRP gave faster relief)
Creaney	2011	PRP vs ABI	Equivocal (equal efficacy)

As the influence of conditioned plasma on TE remains controversial, some studies focused on the biologic effect of injecting PRP versus autologous blood injection (ABI). Creaney et al. reported no difference in pain scores between PRP and ABI, 6 months after injections. They found both methods to be equal in efficacy and recommended them when conservative treatment failed [30•]. Raeissadat et al. also did not observe a significant difference regarding pain, functional scores, and treatment success in all follow-up examinations (respectively 0, 4, 8, 6, 12 months after the procedure) between ABI and PRP [31•]. Chou et al. published a meta-analysis comparing ABI, PRP, and corticosteroid injections. They found no significant difference between PRP and ABI, with both having superior pain scores to corticosteroid injections [32]. PRP may give faster relief as reported by Thanasas et al. In 6 weeks after an injection, patients in the PRP group had significantly lower pain scores versus those in the ABI group [33•]. Studies are detailed in Table 2.

**Table 2:** Comparative studies of PRP in the treatment of elbow pathologies. VAS (Visual Analogue Scale), PRTEE (Patient-Rated Tennis Elbow Evaluation questionnaire), DASH (Disabilities of the Arm, Shoulder and Hand score), ABI (Autologous Blood Injection)

Author	Year	PRP preparation	Study design	No. of cases (*n*)	Outcome scores	Comments
Palacio et al. [27]	2016	60 ml of blood was divided between six 10-ml tubes that contained sodium citrate. These tubes were then subjected to two cycles of centrifugation, under forces of 400*g* and 800*g*, for 10 min. Two thirds of the original volume (platelet-poor plasma) was discarded and only one third of the original blood sample consisted of PRP	PRP vs steroid	PRP (20), steroid (20)	DASH, PRTEE	No significant difference in scores among both groups
Gautam et al. [23]	2015	20 ml of blood was collected in an acid citrate dextrose vacutainer and centrifuged at 1500 rpm for 15 min to separate the blood into layers of red blood cells, buffy-coat of leucocytes, and plasma.	PRP vs steroid	PRP (15), steroid (15)	VAS, DASH, modified Mayo score, hand grip strength, ultrasound	All scores improved significantly from pre-injection to the 6-month follow-up in the PRP and CS groups. However, in the CS group, the scores generally peaked at 3 months and then deteriorated at 6 months indicating recurrence. Higher incidence of reduced tendon thickness and condylar erosions in the CS group
Khaliq [26]	2015	Not described	PRP vs steroid	PRP (51), steroid (51)	VAS	Significant improvement in VAS scores in the PRP group
Lebiedziński et al. [19]	2015	PRP prepared using (Double Syringe System, Arthrex)	PRP vs steroid	PRP (53), steroid (46)	DASH	Mean DASH score at 1 year was significantly better in the PRP group though at 6 weeks and 6 months it was significantly better in the steroid group
Yadav [24]	2015	Patients received a single injection of PRP (1 ml), with absolute platelet count of 1 million platelets/mm^3^ as confirmed by manual counting. PRP was injected into the common extensor origin at the lateral epicondyle of the humerus under aseptic conditions. PRP was prepared under aseptic conditions as per the procedure standardized in the departmental laboratory. A 9001:2000 ISO-certified R-23 centrifuge was used for the purpose of platelet concentration.	PRP vs steroid	PRP (30), steroid (30)	VAS, DASH, grip strength	At 3 months, significant improvement in pain and function scores in the PRP group (Steroid group had better initial pain relief which declined subsequently)
Behera et al. [25]	2015	100 ml blood was collected into an anticoagulant blood bag and centrifuged at 1500 rpm for 15 min. The supernatant fluid was transferred into another blood bag. Leukocytes were filtered out using a filter (Imuguard III-PL, Terumo Penpol, Thiruvananthapuram, India) to obtain leukocyte poor PRP, with the platelet count between 6 and 8 × 105/μL, and the leukocyte count a 3-log reduction. Under ultrasonographic guidance, 3 ml of type-4B PRP and 0.5 ml of calcium chloride was injected.	PRP vs bupivacaine	Leucocyte poor PRP (15), bupivacaine (10)	VAS MMCPIE score, Nirschl score	Significant improvement in all scores in the PRP group at 3 months (Steroid group had better initial results which declined subsequently)
Raeissadat et al. [31]	2014	The PRP processing was done using the Rooyagen kit (made by AryaMabnaTashkhis Corporation, RN: 312569). For preparing 2 ml of PRP with concentration of 4–6 times the average normal values, 20 ml of blood was first collected from the patient’ s upper limb cubital vein using an 18-G needle. Then 2 ml of ACD-A was added to the sample as an anticoagulant. One milliliter of the blood sample was sent for complete blood count. The rest of the sample passed two stages of centrifugation (first with 1600 rpm for 15 minutes for separation of erythrocytes and next with 2800 rpm for 7 min in order to concentrate platelets). The final product was 2 ml of PRP containing leukocytes (leukocyte-rich PRP). The PRP quantification and qualification procedure was performed using laboratory analyzer Sysmex KX 21 and swirling and if approved, the injection was performed	PRP vs autologous blood	Leucocyte-rich PRP (33) ABI (31)	VAS, MAYO score, pressure pain threshold	Although scores of both groups improved over 12 months, no significant difference between PRP vs ABI
Mishra et al. [29]	2014	30 mL of whole blood was drawn from a peripheral vein of each patient. In the PRP group, the blood was mixed with an anticoagulant (ACD-A) and placed into a sterile separator canister (GPS, Biomet Biologics, Warsaw, Indiana). The canister was then placed in a desktop-sized centrifuge and processed for 15 min at 3200 rpm producing type 1A PRP (leukocyte-enriched PRP with platelets 5 times the baseline used in an inactivated manner). The PRP was then removed and buffered to physiological pH using 8.4% sodium bicarbonate to neutralize the acidic ACD-A in the PRP.	Leucocyte-enriched PRP vs active control	PRP (116) Controls (114)	VAS with resisted wrist extension PRTEE scores	Significant pain improvement at 24 weeks compared with controls. No difference in PRTEE scores
Krogh et al. [20]	2013	For the PRP, 27 mL of whole blood was collected into a 30-mL syringe containing 3 mL sodium citrate (anticoagulant) and then placed in a disposable cylinder in a centrifuge for 15 min at a speed of 3.2 (31,000 rpm). Platelets were collected using the Recover GPS II system (Biomet Biologics Inc., Warsaw, Indiana) producing 3 to 3.5 mL of PRP, with a platelet concentration increased on average by 8-fold compared with whole blood. To achieve a physiological pH, the PRP was buffered with 8.4% sodium bicarbonate.	PRP vs steroids vs saline	PRP (20), steroid (20), saline (20)		No significant improvement in pain at 3 months compared to saline or steroids
Gosens et al. [21]	2011	The PRP preparation was done using the Recoversystem (Biomet Biologics, Warsaw, Indiana).	PRP vs steroids	PRP (51), steroid (49)	VAS DASH	Significant improvement in VAS and DASH scores at 2 years of PRP group (DASH scores of steroid groups returned to baseline levels while those of the PRP group improved)
Thanasas [33]	2011	For the PRP preparation, the Biomet GPS III was used. This system uses, under aseptic technique, 27 to 55 mL of autologous peripheral blood with 3to 5 mL of anticoagulant, centrifuges it at 3200 rpm for15 minutes, and finally extracts 3 to 6 mL of PRP. No activator was used.	PRP vs autologous whole blood injection	PRP (14), autologous whole blood (14)	VAS, Liverpool elbow score	Significant pain improvement at 6 weeks. No significant difference in function.
Creaney [30]	2011	Blood was collected using a 21-G needle from the antecubital fossa into an 8.5-ml vacutainer tube with citrate anticoagulant. For patients randomly assigned to plasma injection, the blood was spun in a centrifuge at 2000*g* for 15 min (LC6; Sarstedt, Numbrecht, Germany) and 1.5 ml was siphoned from the buffy coat layer.	PRP vs autologous blood injection	PRP (80), ABI (70)	PRTEE	No significant difference at 6 months. Higher conversion rate to surgery in ABI group
Peerbooms [12]	2010	The patient’s own platelets were collected with the Recover System (Biomet Biologics, Warsaw, Indiana). This device uses a desktop-sized centrifuge with disposable cylinders to isolate the platelet-rich fraction from a small volume of the patient’s anticoagulated blood, drawn at the time of the procedure. Approximately 3 mL PRP was obtained for each patient. The PRP was then buffered to physiologic pH using 8.4% sodium bicarbonate, and bupivacaine hydrochloride 0.5% with epinephrine (1:200000) was added. No activating agent was used.	PRP vs steroid injection	PRP (51), steroids (49)	VAS, DASH	Significant decrease in pain and improvement in VAS and DASH scores at 1 year in PRP group (Steroid group had better initial results which declined subsequently)

Even in the best possible scenario, 5 to 15% patients will require a more aggressive approach [34]. Several studies therefore compared results of PRP treatment with either extracorporeal shockwave (ESW), dry needling, or even surgical treatment. Alessio-Mazzola performed a retrospective comparative study with 2 years follow-up comparing PRP injection with extracorporeal shockwave therapy. In the second group, focal ESW was administered by means of an electromagnetic generator equipped with in-line ultrasound (USG) guidance. The authors found no difference in pain scores and patients’ reported outcomes measured with Roles–Maudsley (RM) score, quick Disabilities of the Arm, Shoulder and Hand (QuickDASH) score, and patient-rated tennis elbow evaluation (PRTEE) [35]. Lim et al. reported that needling when combined with PRP injection gives favorable results versus physiotherapy in pain scores, modified Mayo Clinical Performance scores, and even in MRI imaging [36]. Similarly, beneficial results of PRP injections and concomitant needling were also reported by Gaspar et al. Interestingly, this paper reports sustained good outcomes with PRP at a mean follow-up of over 3 years [37].

There are also two studies comparing PRP injections with open surgical debridement as described many years ago by Nirschl. Ford et al. found no difference in pain relief between these two procedures with a mean follow-up of 315 days for PRP patients and 352 for surgical patients. He has also reported similar return to work rates between the groups [34]. In contrast, better effects with PRP injections were described by Karaduman et al. who reported that even up to 1-year follow-up, TE patients had less pain, higher Mayo Elbow Scores, and stronger grip than those who underwent the Nirschl procedure [38]. The same findings for short and midterm follow-up were found when PRP was compared with arthroscopic TE release. However, improvement after arthroscopic surgical treatment resulted in sustained pain relief with better results for grip strength in more than 2 years follow-up compared to PRP [39]. In a meta-analysis of randomized controlled trials, Fitzpatrick et al. evaluated the outcomes of the PRP groups by preparation method and injection technique in tendinopathy. The aim of this study was to determine the clinical effectiveness of the preparations and to evaluate the effect of controls used in the studies reviewed. Among the results of PRP group good evidence has been found to support the use of a single injection of LR-PRP under ultrasound guidance in tendinopathy over what? [40].

## Golfer’s Elbow

Medial epicondylitis is similar to its lateral counterpart, though less common. Often referred to as “golfer’s elbow” (GE), its incidence is estimated as 1% in a general population [41, 42]. As for TE, a variety of possible treatments have been described in the literature, with no consensus on the ultimate algorithm [43]. However, though it is a well-recognized condition, there is a paucity of studies about PRP administration in GE patients. Varshney et al. published a study in which 83 patients with elbow epicondylitis (63 with TE and 20 with ME) were randomly allocated into two groups: (A) 53 patients treated with corticosteroid injection and (B) 33 patients treated with PRP. He reported 91% improvement in VAS pain score 6 months after PRP injection versus 42.2% in steroid injection group. The major limitation of this study was the lack of reporting separate results for TE and GE. [44]. Tylor and Hannafin suggested that PRP injection may be beneficial for GE, but lack of studies make it difficult to fully assess its utility [45]. This may be the reason why Donaldson et al., who published a treatment algorithm for GE, do not even mention PRP as a treatment modality in their approach [46].

## Ulnar Collateral Ligament Pathology

Ulnar collateral ligament (UCL) insufficiency remains a career-threatening injury for overhead athletes, especially baseball players [47]. A standard conservative treatment protocol that includes physiotherapy and activity modification is reported to be successful in about 42% of cases [48]. Therefore, PRP has gained attention as a possible way of augmenting the conservative treatment of UCL pathology. Dines et al. published outcomes of treatment with PRP injections on 44 baseball players (6 professional athletes) who failed initial conservative treatment. There were 36 pitchers and 8 position players in this study. Thirty-four percent of 44 players had an excellent outcome. Among position players, 4 had an excellent outcome, 3 good outcome, and 1 had a poor outcome. Sixty-seven percent of professional players were capable of returning to play after PRP injection. Generally, in this study, 73% players had a good to excellent outcomes with PRP injection. However, 10 out of 44 patients had poor and 2 had fair outcomes. Interestingly, 100% poor outcomes were observed in patients with distal ligament rupture. They suggested that this treatment may beneficial in young athletes with partial tears who sustain acute trauma [47]. The results of PRP-augmented conservative therapy was also reported by Podesta et al. The authors noted a decrease of medial valgus gapping and return to sport rate of 88% at 12 weeks after treatment of 34 athletes with partial UCL tears. [49]. PRP treatment in partial UCL tears was also evaluated by Deal et al., who included 25 athletes (23 baseball players and 2 softball players). They found that with 2 injections of leukocyte-rich PRP, 96% demonstrated UCL stability 2 weeks after the second injection. Worse results were achieved in patients who underwent previous UCL surgery [50]. Interestingly, a survey of American Shoulder and Elbow members revealed that only 36% of questioned physicians reported using PRP in UCL injuries, and only 16.6% of respondents claimed to use leukocyte-rich PRP [51].

## Distal Biceps Pathology

Distal biceps degeneration is not a common reason for elbow pain. Its description includes pain with elbow flexion and/or supination with a present of intact biceps tendon. Little data exists in examining the results of PRP injection to distal biceps pathology. A small cohort of 6 patients treated with ultrasound guided PRP injections and evaluated with MRI was reported by Barker et al. The mean modified Mayo Elbow Performance Score improved significantly from 68.3 points to 95 points at the mean follow-up of 16 months. Decrease in VAS pain score has also been noted in this cohort and all patients claimed willingness to have another injection if needed. This study has however a major limitation, which is lack of control group [52]. A bigger cohort has been observed by Sanli et al. They also reported significant improvements in VAS pain scores, from median 8 to median 2.5 for active movements. Elbow functional assessment scores also reached significant improvement, reporting a 56% increase in strength. Interestingly, on median follow-up of 47 months, no recurrence of symptoms was observed [53].

## Distal Triceps Pathology

We found only one case report of a 47-year-old male with a partial distal triceps tendon rupture who was treated with PRP injection and consecutive physiotherapy. On reexamination, performed 2 weeks after injection and before a course of physiotherapy, a decrease of pain was noted. At 4 weeks after injection, an increase in strength with elbow extension was noted from 3/5 to 4/5 [54]. Given the incidental report and no control, it is hard to recommend injection at this time for a standard treatment in distal triceps pathology. However, growing interest in biologic treatment may provide further study in the future.

## Conclusions

Interest in biologic treatments of soft tissue pathologies has increased over the last several decades. The number of published studies on PRP has exceed 14,000 in PubMed and there is still no consensus on whether it should be employed in soft tissue pathology management or not. The methodological challenges regarding concentration, preparation, and activation continue to limit the ability to draw definitive conclusions from the literature.

Regarding elbow pathologies, PRP injection in tennis elbow seems to be the best-studied intervention. However, although numerous original studies suggest potential benefit of PRP, especially when compared to steroid injections, available systematic reviews and meta-analyses leave the physician with inconclusive evidence. Therefore, not only more studies on larger cohorts and with longer follow-up are required but also standardization in the method of preparing PRP, deciding on leukocyte-rich or leukocyte-poor preparations, PRP with or without activation, and uniformity in pain and function evaluation scores are some of the areas which need to be agreed upon by collaborating international working groups before meaningful recommendations can be made regarding the efficacy of PRP.

Incidental reporting of treating other than TE elbow conditions makes it impossible to make a recommendation either for or against PRP. Therefore, we suggested caution in discussing this possibility with patients.
